# Decreased myelin content of the fornix predicts poorer memory performance beyond vascular risk, hippocampal volume, and fractional anisotropy in nondemented older adults

**DOI:** 10.1007/s11682-021-00458-z

**Published:** 2021-02-26

**Authors:** Katherine J. Bangen, Lisa Delano-Wood, Sean C. L. Deoni, Alexandra L. Clark, Nicole D. Evangelista, Samantha N. Hoffman, Scott F. Sorg, Sophia Holmqvist, Jessica Osuna, Alexandra J. Weigand, Amy J. Jak, Mark W. Bondi, Melissa Lamar

**Affiliations:** 1 Research Service, VA San Diego Healthcare System, San Diego, CA USA; 2 Department of Psychiatry, University of California, San Diego, 9500 Gilman Drive, Mail Code 151B, La Jolla, CA 92093-9151 USA; 3 Psychology Service, VA San Diego Healthcare System, San Diego, CA USA; 4 Department of Pediatrics, Brown University, Providence, RI USA; 5 Center for Cognitive Aging and Memory, Department of Clinical and Health Psychology, McKnight Brain Institute, College of Public Health and Health Professions, University of Florida, Gainesville, FL USA; 6 San Diego Joint Doctoral Program in Clinical Psychology, San Diego State University/University of California, San Diego, CA USA; 7 Rush Alzheimer’s Disease Center, Department of Psychiatry and Behavioral Sciences, Rush University Medical Center, Chicago, IL USA

**Keywords:** Aging, Memory, Neuropsychology, Human brain imaging, Myelin

## Abstract

Alterations to cerebral white matter tracts have been associated with cognitive decline in aging and Alzheimer’s disease (AD). In particular, the fornix has been implicated as especially vulnerable given that it represents the primary outflow tract of the hippocampus. Despite this, little work has focused on the fornix using a potential early marker of white matter degeneration—myelin water fraction (MWF; an in vivo marker of myelin content). Therefore, we sought to (1) clarify associations between MWF in the fornix and memory functioning, and (2) examine whether fornix MWF relates to memory performance above and beyond hippocampal volume and conventional imaging measures of white matter that may not be as specific to alterations in myelin content. Forty nondemented older adults (mean age = 72.9 years) underwent an MRI exam and neuropsychological assessment. Multicomponent driven equilibrium single pulse observation of T1 and T2 (mcDESPOT) was used to quantify fornix MWF and diffusion tensor imaging (DTI) was used to measure fornix fractional anisotropy (FA). Adjusting for age, sex, education, and vascular risk factors, linear regression models revealed that, lower fornix MWF was significantly associated with poorer memory functioning (*β* = 0.405, *p* = .007) across our sample of older adults. Notably, fornix MWF remained a significant predictor of memory functioning (*β* = 0.380, *p* = .015) even after adjusting for fornix DTI FA and hippocampal volume (in addition to the above covariates). Given the observed associations between myelin and memory in older adults without dementia, MWF may be a useful early marker of dementia risk.

**Supplementary Information:**

The online version contains supplementary material available at 10.1007/s11682-021-00458-z.

## Introduction

There is growing interest in elucidating patterns of white matter (WM) alterations in aging and Alzheimer’s disease (AD) risk (Alm et al. 2019; Bangen et al. 2018, 2020). This work aims to identify biomarkers associated with memory decline that may have relevance in cognitive aging and in the development of neurodegenerative disease such as AD. Prior magnetic resonance imaging (MRI)-based studies suggest that WM alterations including WM lesions, and reduced integrity of medial temporal lobe (MTL) WM pathways are sensitive markers of early decline in aging and AD (Bangen et al. 2020; Bartzokis 2011; Stebbins and Murphy 2009). Further, WM damage has been to shown to predict cognitive decline independently of cerebrospinal fluid (CSF) measures of amyloid and tau or MRI-derived hippocampal volume (Brickman et al. 2012; Selnes et al. 2013).

The fornix—the principal efferent tract of the hippocampus (Teipel et al. 2016)—plays a role in episodic memory, which declines in normal aging (Head et al. 2008) and is also typically the first cognitive domain affected in AD (Salmon and Butters 1992). Diffusion tensor imaging (DTI) studies show that fornix WM microstructure relates to episodic memory in cognitively normal older adults (Metzler-Baddeley et al. 2011) and those at risk for AD (Mielke et al. 2012). Notably, fornix microstructural changes are detectable in presymptomatic carriers of familial AD mutations years before they develop cognitive dysfunction and when gray matter volume is still preserved (Ringman et al. 2007), suggesting fornix integrity may be useful in predicting memory decline.

Neuropathologic studies in AD document loss of myelin lamellae but relatively normal-appearing axoplasm, suggesting primary demyelination in AD (Terry et al. 1964). Dovetailing with this finding, neuropathologic studies also show that reduced myelin density, but not axonal density, contribute to rarefication of WM in cognitively normal older adults (Murray et al. 2012). Therefore, MRI techniques specifically assessing myelin may elucidate microstructural mechanisms contributing to WM changes in aging and AD (Lamar et al. 2014). To date, DTI has been the most widely used MRI method to quantify WM microstructure, which may relate, in part, to its accessible protocols on clinical scanners, and availability of free and user-friendly software for data analysis (Lebel and Deoni 2018). Although DTI may detect WM microstructural abnormalities, potential weaknesses include that conventional DTI indices (e.g., fractional anisotropy [FA]) may reflect neuropathology of many forms including axonal size, density, and configuration (e.g., crossing fibers) (Beaulieu 2002); are not specific to myelin; and may be confounded by inflammation (Taber and Hurley 2013).

Multicomponent-driven equilibrium single pulse observation of T1/T2 (mcDESPOT), is a promising technique for quantification of myelin content (Deoni et al. 2008) and may provide complementary information to that obtained with DTI. mcDESPOT takes advantage of differential T1 and T2 relaxation times of unbound and trapped water in order to visualize the intra- and extra-cellular water within the myelin sheath and calculates myelin water fraction (MWF), an indicator of myelin volume. Not without its own limitations (e.g., complex postprocessing and low contrast-to-noise ratios in regions with low myelin content (Prasloski et al. 2012; Uddin et al. 2019), MWF is confirmed to be myelin-specific. For example, MWF measures correlate highly with histological measures of myelin density (Laule et al. 2006) and are insensitive to inflammation (Gareau et al. 2000) (See Lamar et al. 2014 for review).

mcDESPOT has begun to be applied in aging (Bouhrara et al. 2020); however, to our knowledge, no studies have investigated associations between mcDESPOT MWF and memory among nondemented older adults. Although T1-weighted hippocampal volume, fornix DTI FA, and fornix MWF all likely contribute to variability in memory performance, given that myelin has been less studied, we focused on MWF. We sought to (1) clarify associations between fornix MWF and memory, and (2) examine whether fornix MWF relates to memory above and beyond hippocampal volume and conventional measures of WM integrity that may not be specific to myelin content. We hypothesized that reduced fornix MWF would be associated with lower memory performance, even after adjusting for hippocampal volume and fornix DTI FA.

## Methods

### Participants

Forty older adults were recruited from ongoing aging studies at the University of California, San Diego and the VA San Diego Healthcare System (VASDHS). Exclusion criteria included history of dementia, stroke, neurologic disease, head injury with cognitive sequelae, or major psychiatric disorder. Participants were not excluded based on any other factors including vascular risk burden. All participants provided informed consent. The VASDHS Institutional Review Board approved the protocol.

### Clinical assessment

Participants underwent clinical interview assessing medical and psychiatric history; brachial artery blood pressure measurement; and neuropsychological assessment. Participants were classified as having normal cognition (n = 34) or mild cognitive impairment (MCI) (n = 6; specifically, 4 with amnestic MCI and 2 with nonamnestic MCI) based on Jak/Bondi neuropsychological criteria (see Supplemental Material for criteria details).

Vascular risk was quantified using the updated Framingham Stroke Risk Profile (FSRP), which better predicts risk compared to previous versions (Dufouil et al. 2017). The updated FSRP provides sex-corrected scores based on age, systolic blood pressure, diabetes, cigarette smoking, cardiovascular disease, atrial fibrillation, and antihypertensive medication use.

### Cognitive composite variable construction

Episodic memory was assessed by California Verbal Learning Test-Second Edition (CVLT-II) (Delis et al. 2000) and Wechsler Memory Scale-Revised (WMS-R) Visual Reproduction (Wechsler 1987). To reduce statistical comparisons, a memory composite variable was computed including the following six items: CVLT-II total learning trials 1–5, short delay free recall, long delay free recall, and total recognition discriminability; and WMS-R Visual Reproduction immediate and delayed recall. Demographically-corrected scores were converted into z-scores and averaged to create composite scores (See Supplemental Material for information on demographic corrections). Cronbach’s alpha was 0.92, indicating high internal consistency for the memory composite.

Although episodic memory performance was of primary interest, to address convergent versus divergent validity of the associations between cognition and MWF, we calculated an executive function composite score. Executive function measures included Trail Making Test, Part B (Heaton et al. 2004); Delis-Kaplan Executive Function Test (D-KEFS) Letter Fluency total score; D-KEFS Color-Word Interference inhibition and inhibition/switching scores (Delis et al. 2001); and the Wisconsin Card Sorting Test–64 Card Version (Kongs et al. 2000) number of categories and perseverative responses. Demographically-corrected scores were converted into z-scores and averaged to create a composite score. Cronbach’s alpha was 0.67 for the executive function composite suggesting acceptable internal consistency.

### MRI data acquisition

MRI was performed on a GE 3T scanner. A T1-weighted high-resolution anatomical scan was collected using a Fast Spoiled Gradient Recall acquisition (172 1mm contiguous sagittal slices, field of view [FOV] = 25 cm, repetition time [TR] = 8ms, echo time [TE] = 3.1ms, flip angle = 12, inversion time [TI] = 600ms, 256 × 192 matrix, Bandwidth = 31.25 kHz, frequency direction = S-I, NEX = 1).

DTI data were acquired using dual spin-echo EPI acquisition (FOV = 240mm, slice thickness = 3mm, 128 × 128 matrix, in-plane resolution = 1.875 × 1.875, TR = 8000ms, TE = 93ms). Thirty-four slices were acquired with 61 diffusion directions distributed on the surface of a sphere in conjunction with the electrostatic repulsion model (Jones et al. 1999) and b value of 1500s/mm^2^. We included one T2-weighted image with no diffusion (b = 0). Field maps were collected to reduce distortions due to lack of magnetic field homogeneity.

For the mcDESPOT sequence, we acquired a series of spoiled gradient recalled echo (SPGR; TR = 5.3ms, TE = Min Full, flip angle = 18, FOV = 24.0) and T2/T1-weighted balanced steady-state free precession (SSFP) data over a range of flip angles (Deoni 2011). To correct for B1 inhomogeneities, we collected an inversion-recovery prepared SPGR (IR-SPGR) scan (TR = 5.3ms, TE = Min Full, flip angle = 5, FOV = 24.0). We collected SSFP phase 180 (TE = Min Full, flip angle = 60, field of view = 24.0) and SSFP phase 0 (TE = Min Full, flip angle = 60, FOV = 24.0) with two phase-cycling patterns to correct for main magnetic field (B0) off-resonance effects.

See Supplemental Material for MRI data processing methods.

### Statistical analyses

Hierarchical linear regressions examined whether fornix MWF was associated with memory after adjusting for covariates. For Model 1, we entered age, sex, education, and FSRP into Block 1, all variables that are risk factors for cognitive decline. For Model 2, we entered all Model 1 variables plus hippocampal volume and fornix DTI FA into Block (1) For both Models 1 and 2, fornix MWF was entered into Block (2) The DTI scan was not acquired for one participant so analyses including DTI indices included 39 participants.

We included FA in primary analyses, given this is the most commonly used DTI metric, however, given that changes in radial diffusivity (RD) have been purported to signify loss of myelin integrity (Song et al. 2002), we performed secondary analyses substituting fornix RD for fornix FA. Also, in secondary analyses, we re-ran primary Models 1 and 2 with the additional covariate of the logarithmic transformation of total volume of FreeSurfer-derived white matter signal abnormalities (WMSA). Natural log transformation was used as raw value distributions of WMSA were positively skewed. To examine the specificity of fornix MWF to memory, we re-ran analyses with the executive functioning composite score (instead of the memory composite score) as the dependent variable. Finally, in a sensitivity analysis, we re-ran primary models restricting the sample to cognitively normal participants (n = 34). Multicollinearity of independent variables was assessed and all variance inflation factor values were less than 3. Significance levels of 0.05 were used for all tests. Analyses were conducted with Statistical Package for the Social Sciences (SPSS) version 26.

## Results

### Demographics and clinical characteristics

Sample characteristics are presented in Table 1. On average, the sample was approximately 73 years, well-educated, and had low vascular risk burden. In addition, the mean estimated verbal IQ of the sample (123 ± 2.99) was approximately 1.5 standard deviations higher than the general population mean of 100 and the mean score on a measure of global cognitive functioning was unimpaired (i.e., Dementia Rating Scale total score of 140).

**Table 1:** Sample characteristics

Variable	Mean (SD, Range)
Demographics	
Age, years	72.93 (5.93, 55–84)
Education, years	16.85 (1.79, 12–20)
Sex (% Female)	65.00
Ethnicity (% White)	90.00
Clinical and Vascular Risk Variables	
Cognitive Status	
% with Normal Cognition	85.00
% with Mild Cognitive Impairment	15.00
FSRP	8.37 (5.95, 0.57—21.86)
GDS	4.00 (3.76, 0—11)
Neuropsychological Measures	
DRS Total Score	140.15 (3.75, 127—144)
ANART Estimated VIQ	123.46 (2.99, 118—128)
Episodic Memory*	0.00 (0.83, -1.84—1.7)
Executive Functioning*	0.00 (0.63, -1.24—1.14)

* Composite scores computed as the average of demographically adjusted z-scores of measures within that cognitive domain

** Hippocampal volume was normalized by dividing by total intracranial volume and then multiplying by 1,000

Abbreviations: FSRP = updated Framingham Stroke Risk Profile (Dufouil et al. 2017); GDS = Geriatric Depression Scale; DRS = Dementia Rating Scale; ANART = American National Adult Reading Test; VIQ = verbal intelligence quotient

### Association between fornix MWF and memory

**Fig. 1:**
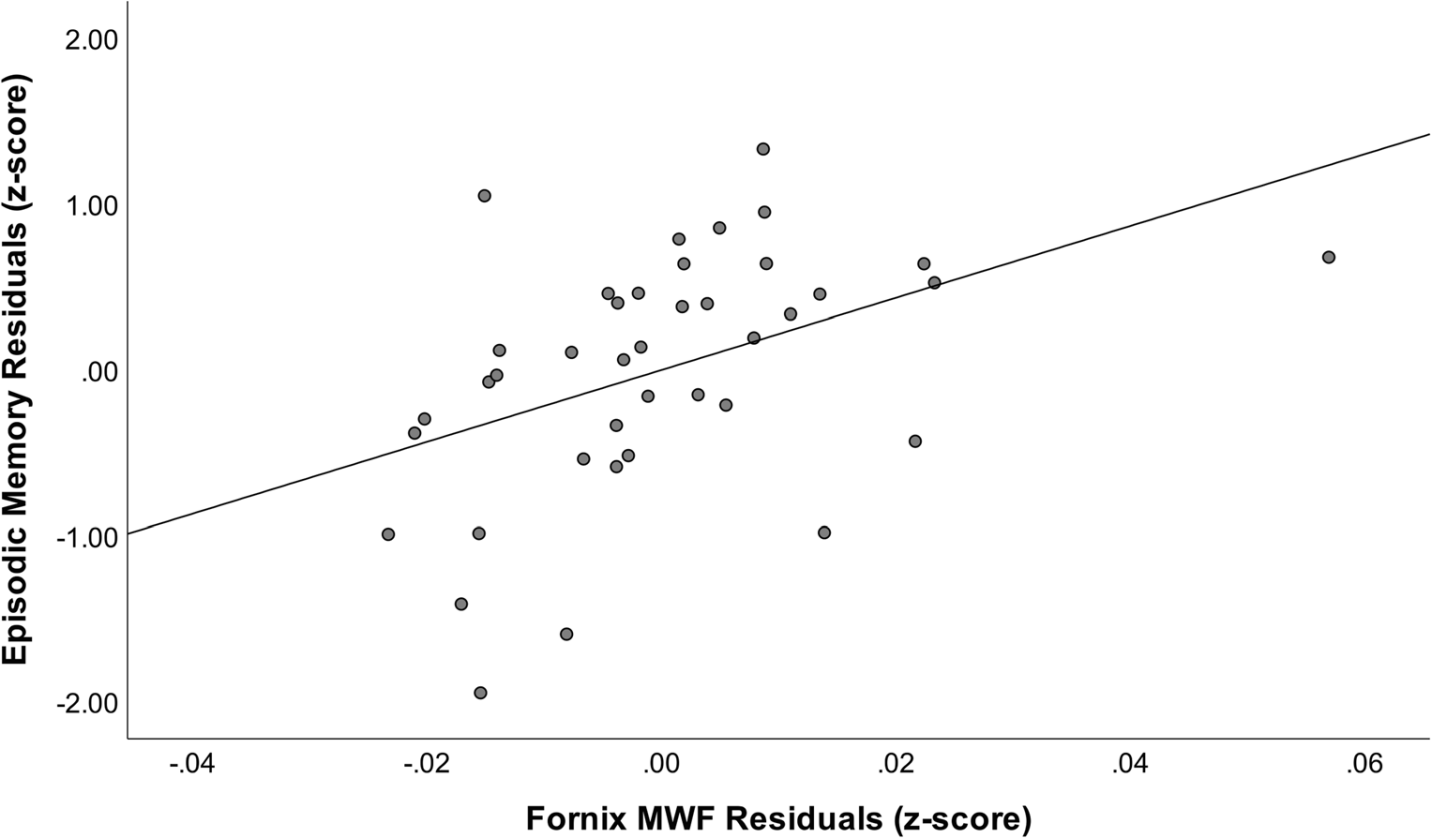
Partial regression plot for association between episodic memory performance and fornix MWF adjusting for age, education, sex, and FSRP. Abbreviations: MWF = myelin water fraction; FSRP = Framingham Stroke Risk Profile

In Model 1, age, education, sex, and FSRP were entered into Block 1 which explained 20.8 % of the variance in memory performance. A significant increase in the amount of variance in memory was observed when the fornix MWF was added as Block 2 of the model, with the overall model explaining approximately 37 % of the variance (Δ*R*^*2*^ = 0.157, Δ*F*(1,34) = 8.387, *p* = .007). Lower fornix MWF was associated with poorer memory performance (β = 0.405, p = .007; Fig. 1). See Table 2. There was one fornix MWF outlier. Excluding that participant did not change the pattern or significance of results.

**Table 2:** Multiple hierarchical linear regression models for association of fornix MWF and memory functioning adjusting for demographics and vascular risk burden

	Variable	B	*SE*	β	*t*	*p*	F	R^2^	Δ R^2^	Block p
Block 1							2.298	0.208		0.078
	Age	0.018	0.026	0.128	0.679	0.502				
	**Education**	.**195**	.**077**	.**423**	**2.530**	.**016**				
	Sex*	0.315	0.271	0.184	1.160	0.254				
	FSRP	0.041	0.026	0.297	1.161	0.115				
Block 2							**3.904**	.**365**	.**157**	.**007**
	Age	0.014	0.024	0.098	0.569	0.573				
	**Education**	.**180**	.**070**	.**390**	**2.561**	.**015**				
	Sex*	0.427	0.250	0.249	1.709	0.097				
	FSRP	0.044	0.023	0.314	1.880	0.069				
	**Fornix MWF**	**21.688**	**7.489**	.**405**	**2.896**	.**007**				

Abbreviations: FSRP = updated Framingham Stroke Risk Profile (Dufouil et al. 2017); MWF = myelin water fraction

* Note males served as the reference group

Statistically significant (p < .05) results appear in bold font

**Fig. 2:**
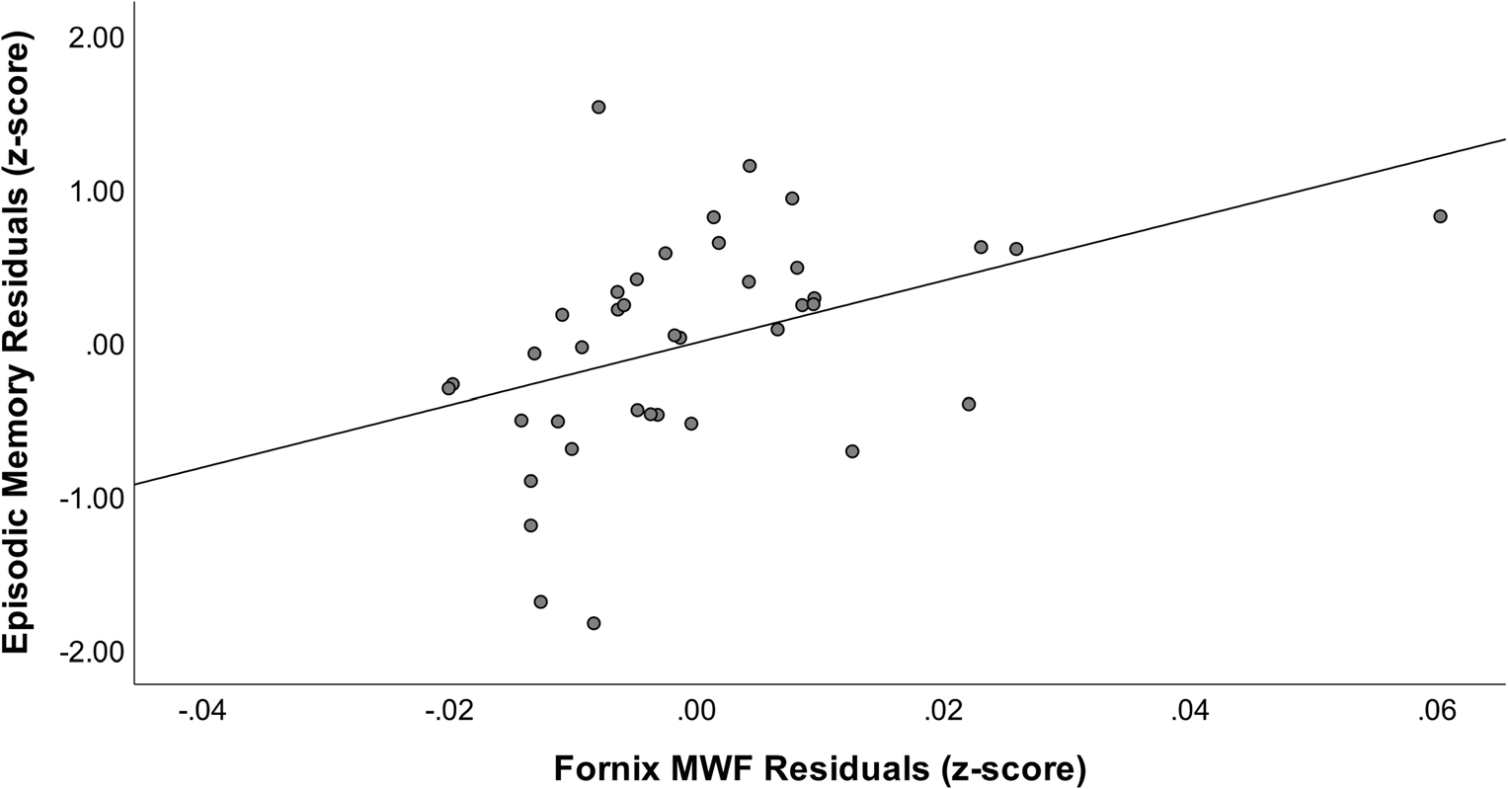
Partial regression plot for association between episodic memory performance and fornix MWF adjusting for age, education, sex, FSRP, normalized hippocampal volume, and fornix DTI FA. Abbreviations: MWF = myelin water fraction; FSRP = Framingham Stroke Risk Profile; DTI = diffusion tensor imaging; FA = fractional anisotropy

For Model 2, age, education, sex, FSRP, hippocampal volume, and fornix DTI FA were entered into Block 1, and fornix MWF was entered into Block 2. Results are presented in Table 3. Block 1 explained 25.7 % of the variance in memory performance. A significant increase in the amount of variance in memory was observed when the fornix MWF was added as Block 2 of the model, with the overall model explaining approximately 39 % of the variance (Δ*R*^*2*^ = 0.130, Δ*F* (1,31) = 6.600, *p* = .015). Lower fornix MWF (β = 0.380, p = .015; Fig. 2; Table 3) but not FA (β=-0.058, p = .786) was associated with poorer memory performance. Excluding the aforementioned outlier did not change the results. Results remained similar when models were re-run substituting fornix RD in place of fornix FA. That is, in this model, fornix MWF (β = 0.381, p = .014) but not RD (β = 0.088, p = .681) was significantly associated with memory performance.

**Table 3:** Multiple hierarchical linear regression models for association of fornix MWF and memory functioning adjusting for demographics, vascular risk burden, hippocampal volume, and fornix DTI FA

	Variable	B	*SE*	β	*t*	*p*	F	R^2^	Δ R^2^	Block p
Block 1							1.847	0.257		0.121
	Age	0.028	0.032	0.199	0.859	0.396				
	**Education**	.**182**	.**079**	.**394**	**2.316**	.**027**				
	Sex*	0.150	0.295	0.087	0.508	0.615				
	FSRP	0.042	0.027	0.300	1.546	0.132				
	Hippocampal Volume	0.378	0.314	0.248	1.202	0.238				
	Fornix DTI FA	0.246	3.190	0.018	0.077	0.939				
Block 2							**2.803**	.**388**	.**130**	.**022**
	Age	0.015	0.030	0.109	0.504	0.618				
	**Education**	.**171**	.**073**	.**369**	**2.346**	.**026**				
	Sex*	0.292	0.275	0.264	1.568	0.128				
	FSRP	0.046	0.025	0.334	1862	0.072				
	Hippocampal Volume	0.313	0.291	0.206	-1.077	0.290				
	Fornix DTI FA	− 0.812	2.2972	− 0.058	− 0.273	0.786				
	**Fornix MWF**	**20.246**	**7.881**	.**380**	**2.569**	.**015**				

Abbreviations: MWF = myelin water fraction; DTI = diffusion tensor imaging; FA = fractional anisotropy; FSRP = updated Framingham Stroke Risk Profile (Dufouil et al. 2017)

* Note males served as the reference group; Statistically significant (*p* < .05) results appear in bold font

See Supplemental Material file for results of secondary analyses.

## Discussion

Our results suggest that fornix MWF is associated with episodic memory performance independent of important confounds and imaging-related variables. These findings remained when analyses were restricted to those participants with normal cognition. Although hippocampal measures have been studied in the context of age- and AD-related memory changes, our findings and those of others (Fletcher et al. 2013; Gold et al. 2010) suggest that the fornix relates to memory performance above and beyond hippocampal volume. Notably, fornix MWF was associated with episodic memory but not executive functioning. This is consistent with the well-established role of the medial temporal lobe (MTL) in episodic memory and suggests specificity of the association of fornix integrity and cognitive functions. Our findings extend previous DTI work suggesting that fornix myelin may be a promising biomarker for early MTL changes in aging and AD risk (Fletcher et al. 2013; Gold et al. 2010) to mcDESPOT MWF.

Our results suggest that myelin-specific measures explain variance in memory performance not explained by DTI-derived FA and T1-derived hippocampal volume. Previous studies also suggest that myelin mapping appears to contribute complementary information relative to traditional WM imaging. For example, a previous study of 61 healthy adults across a wide age range (18–84 years) (Arshad et al. 2016) found that, age differences in myelin content showed a parabolic (inverted U) relationship across the adult lifespan, in agreement with the pattern observed in postmortem work and other *in vivo* imaging studies (Bouhrara et al. 2020). A recent study showed that this nonlinear relationship between myelin content and age was consistent across all regions examined, suggesting that myelination continues until middle age and then decreases at older ages (Bouhrara et al. 2020). Taken together, results support the retrogenesis model of myelin breakdown in normal and pathological brain aging (Reisberg et al. 1999). Specifically, the retrogenesis model states that pathways that myelinate later in normal development, e.g., limbic pathways that include the fornix, breakdown earlier with aging than pathways that myelinate early (Bartzokis 2004). Furthermore, although MWF and DTI measures are indicators of brain microstructure, each of these indices are sensitive to different combinations of tissue characteristics (Uddin et al. 2019) as evidenced by our results and others. Indeed, Arshad and others found that DTI-derived indices that are often cited as proxies for myelin (i.e., FA and RD) were not related to myelin content in any ROIs with the exception of RD in the splenium, suggesting that individual variation in myelin content was not captured by FA or RD (Arshad et al. 2016).

WM changes in AD are postulated to involve multiple biological substrates including non-focal, mild ischemia that results in a gradual loss of oligodendrocytes and myelin, Wallerian degeneration secondary to gray matter atrophy, and/or axonal injury resulting from the cytotoxic effects of β-amyloid on oligodendrocytes (Desai et al. 2010; Englund et al. 1988; Fletcher et al. 2013; Sjobeck et al. 2005). Accumulation of hyperphosphorylated tau may also disrupt WM microstructure as tau binds to and stabilizes microtubules, the latter which is critical for maintaining structural integrity and axonal transport (Shahani and Brandt 2002). Longitudinal myelin-focused studies that also examine CSF β-amyloid and tau measures are needed to elucidate these potential biological pathways. Future studies with a wider range of vascular risk may help further disentangle possible cerebrovascular substrates. Our sample was medically healthy as evidenced by their low prevalence of medical factors considered in the FSRP including cardiovascular disease (12.5 %), diabetes (0 %), and current smoking (5 %) compared to the general population over age 65 (24–37 %, 22–23 %, and 5–11 %, respectively) (Villarroel et al. 2019). However, we have previously shown that cerebrovascular pathology has an additive effect with AD pathology on cognitive impairment, even in patients with relatively mild cerebrovascular changes (Bangen et al. 2015).

Our memory composite included both verbal and visual memory indices, which we consider a strength as it more broadly represents episodic memory than if we included only verbal or visual indices. In addition, neither age, sex, nor hippocampal volume associated with memory performance. Although the hippocampus plays an important role in episodic memory, other studies have found no statistically significant direct relationship between hippocampal volume and episodic memory performance in nondemented older adults but an interaction whereby there was a positive association between hippocampal volume and episodic memory only among individuals with lower levels of cognitive reserve (Vuoksimaa et al. 2013). Nondemented participants in the present study, on average, were highly educated and had high premorbid intellectual function (suggesting higher cognitive reserve); therefore, the association between hippocampal volume and memory performance may have been attenuated. In addition, it is also possible that hippocampal volume loss needs to reach a specific threshold of atrophy before associations are apparent and future studies should examine this assertion. For example, associations between hippocampal volume and memory may be stronger in a sample with lower cognitive reserve and/or more prominent risk factors for AD.

Limitations of our study include cross-sectional design and relatively small sample size. The sample also lacked diversity in race and ethnicity. Future studies with larger, more diverse samples are warranted to confirm our findings. It is possible that whole brain, voxelwise statistical analyses may have yielded brain-cognition associations in regions beyond the *a priori* ROIs examined in the present study. In addition, some may argue that additional adjustment for WMSA is needed given our focus on white matter integrity in an older age cohort more prone to such WM alterations; however, further adjusting for FreeSurfer-derived WM signal abnormalities did not change reported results (see Supplemental Material). Also, given the small number of participants with MCI, we could not perform analyses comparing cognitive groups (MCI versus normal cognition) or MCI subtypes or profiles in terms of MWF, which is an important future direction to further establish the use of MWF as a marker of dementia risk. In addition, the incorporation of intercompartmental exchange in mcDESPOT signal modeling reduces the stability of MWF estimation (West et al. 2019). These issues remain a challenge across all quantitative MRI studies of myelin content, and work is ongoing to further refine existing methods and develop new techniques for improving MWF estimation (Bouhrara et al. 2018; Bouhrara and Spencer 2016). Finally, there is a potential for partial volume bias in MWF calculations. The fornix is a small fiber pathway and may be prone to partial volume effects given its close proximity to CSF. To help mitigate these potential effects of partial voluming and atrophy, we multiplied segmented WM masks by ROI masks to ensure inclusion of only WM voxels in the MWF analyses and adjusted for hippocampal volume. Given that effects of the fornix on memory remained significant even after adjusting for potential influences of hippocampal volume differences across participants, it is unlikely that results can be explained by hippocampal atrophy.

## Conclusions

Our results suggest that fornix MWF relates to episodic memory independent of demographic characteristics, vascular risk, hippocampal volume, and fornix DTI FA in nondemented older adults. Taken together, our findings suggest that fornix myelin may be a promising biomarker in aging and AD risk.

## Supplementary Information

ESM 1 (DOC 201 KB)

## Declarations

The research protocol was approved by the VA San Diego Healthcare System Institutional Review Board. All participants provided written informed consent.
