# Supplementary material for: Decreased myelin content of the fornix predicts poorer memory performance beyond vascular risk, hippocampal volume, and fractional anisotropy in nondemented older adults
Source: Brain Imaging Behav. 2021 Feb 26;15(5):2563–71. doi: 10.1007/s11682-021-00458-z (PMC8500888; doi:10.1007/s11682-021-00458-z)
Supplement: Supplementary file 1 — (DOC 201 KB) [file 11682_2021_458_MOESM1_ESM.doc]

**Supplemental Materials**

**Jak/Bondi MCI criteria:**

Using Jak/Bondi mild cognitive impairment (MCI) criteria (Bondi et al., 2014; Jak et al., 2009), participants were considered MCI if they performed >1 SD below the demographically adjusted mean on two or more neuropsychological measures within the same cognitive domain. Participants classified as MCI were further categorized as amnestic MCI if memory was impaired based on the above criteria and nonamnestic MCI if memory was intact but non-memory domains (e.g., executive functioning, language) were impaired. Participants who did not meet criteria for MCI, were classified as cognitively normal.

**Demographic Corrections of Neuropsychological Measures:**

Normative data was drawn from published norms for all tests. All tests were age-corrected with additional demographics used in published norms as follows: The standardized scores for the California Verbal Learning Test-Second Edition (CVLT-II) further corrected for sex (Delis, Kramer, Kaplan, & Ober, 2000) as did Trails B published norms. Trails B published norms also included ethnicity and education (Heaton, Miller, Taylor, & Grant, 2004). The Wisconsin Card Sorting Test – 64 card version (WCST-64) scores also corrected for education (Kongs, Thompson, Iverson, & Heaton, 2000).

**MRI Data Processing**

**High resolution T1-weighted images.** T1-weighted images were processed using FreeSurfer 6.0 (Dale, Fischl, & Sereno, 1999; Fischl et al., 2002) and all volumetric data was derived from FreeSurfer. FreeSurfer output was visually inspected and, when necessary, manually edited. Hippocampal volume was normalized by dividing by total intracranial volume. See Figure S1 for example of hippocampal ROI. Total volume of white matter signal abnormalities (WMSA), which are thought to reflect small vessel cerebrovascular disease, was also obtained from FreeSurfer.


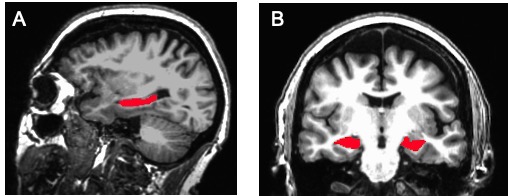


Figure S1. FreeSurfer derived segmentation of the hippocampus used to calculate hippocampal volume shown in sagittal (A) and coronal (B) views for one participant

**DTI processing.** DTI preprocessing used the Oxford Centre for Functional Magnetic

Resonance Imaging of the Brain (FMRIB) Software Library (FSL) (Smith et al., 2004). Two field maps were used to unwarp EPI acquisitions. Images were motion corrected and visually inspected for quality control purposes. FSL’s *dtifit* program was used for voxel-by-voxel calculation of diffusion eigenvalues and to calculate FA, a directional measure of diffusion ranging from 0 (isotropic diffusion) to 1 (perfectly anisotropic diffusion). A nonlinear registration was performed to align all FA images to a target 1x1x1mm standard space (FMRIB58_FA standard-space image). The target was than affine-transformed to 1x1x1mm MNI152 space. The nonlinear transform to the target and the affine transform to MNI152 space were applied to each participant’s FA image, resulting in a 1x1x1mm MNI152 standard space version of each participant’s FA image. The transformed FA images were then averaged to create a mean FA image. The mean FA image was thinned to create a mean FA skeleton, which identifies the voxel with the highest FA as the center of the tract. The resulting mean FA skeleton reflects the central portion of fiber pathways that are common to all participants. To exclude voxels that are primarily gray matter or cerebrospinal fluid, a threshold FA value of .2 was applied. Each individual participant's aligned FA data was probabilistically projected onto this skeleton. The same perpendicular tract direction that was used to create the original mean FA skeleton was used to search each individual's FA image to find the maximum FA value and assign this value to the skeleton voxel. Each participant’s FA skeleton should contain the center of their unique WM tracts, adjusted to the alignment of the group (Stricker et al., 2009). The same transformations derived for the FA maps were applied to the radial diffusivity (RD) maps for use in secondary analyses. The fornix ROI was identified using the ICBM-DTI-81 stereotaxic WM parcellation map (Mori et al., 2008) an applied to the FA and RD maps.

**mcDESPOT processing.** MWF maps were derived by fitting SPGR and bSSFP data to a three-pool model that includes two exchanging water pools (myelin water and water inside and outside the axon) and a third non-exchanging free water pool (Deoni, Dean, O'Muircheartaigh, Dirks, & Jerskey, 2012). Voxel dimensions of the MWF map was approximately 1.7mm^3^.

FSL was used for postprocessing mcDESPOT data. Non-brain voxels were removed from images using Brain Extraction Tool (BET). We linearly, and then non-linearly, registered mcDESPOT target and myelin volume fraction images to the MNI152 T1 2mm resolution brain template via FMRIB's Linear Image Registration Tool (FLIRT) (Jenkinson, Bannister, Brady, & Smith, 2002), followed by FMRIB’s Nonlinear Image Registration Tool (FNIRT). We applied FSL’s Automated Segmentation Tool (FAST) to segment T1-weighted images into WM, gray matter, and cerebrospinal fluid compartments. We then applied the resulting FNIRT transforms to the WM segmentation and myelin volume fraction masks and then multiplied segmented WM masks by ROI masks to ensure inclusion of WM voxels only. As with DTI data, the fornix ROI applied to mcDESPOT data was identified using the ICBM-DTI-81 stereotaxic WM parcellation map. See Figure S2.


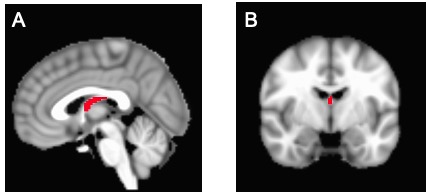


Figure S2. Fornix region of interest overlaid on the MNI152 template shown in sagittal (A) and coronal (B) views. The fornix ROI was applied to mcDESPOT MWF maps and DTI FA and RD maps

**Secondary Analyses Additionally Adjusting for WMSA**

When we ran a model adjusted for demographics, FSRP, hippocampal volume, fornix DTI FA, and total (log transformed) WMSA volume in Block 1, and fornix MWF in Block 2, all results remained similar as those from primary models. That is, fornix MWF was significantly associated with memory (β=.381, p=0.017). Age, education, sex, FSRP, hippocampal volume, fornix FA, and WMSA volume were not associated with memory performance (all p’s>.05).

**Secondary Analyses Examining Associations between Fornix MWF and Executive Functioning**

As hypothesized, in Model 1, when age, education, sex, and FSRP were entered into Block 1 and fornix MWF was entered into Block 2, MWF was not associated with executive functioning performance (β=-.014, p=.929). Similarly, in Model 2, when age, education, sex, FSRP, hippocampal volume, and fornix DTI FA were entered into Block 1 and fornix MWF was entered into Block 2, MWF again was not associated with executive functioning performance (β=-.050, p=.754).

**Sensitivity Analyses in Cognitively Normal Participants**

When we reran our primary analyses restricting the sample to the cognitively normal participants only (n=34), findings were somewhat attenuated compared to the analyses including the entire sample although fornix MWF remained significantly associated with memory performance. See Tables S1 and S2.

Table S1. Multiple Hierarchical Linear Regression Models for Association of Fornix MWF and Memory Functioning Adjusting for Demographics and Vascular Risk Burden Among Cognitively Normal Participants (n = 34)

|  | Variable | B | *SE* | β | *t* | *p* | F | R^2^ | Δ R^2^ | Block p |
| --- | --- | --- | --- | --- | --- | --- | --- | --- | --- | --- |
| **Block 1** |  |  |  |  |  |  | 2.297 | .241 |  | .083 |
|  | Age | .029 | .021 | .287 | 1.370 | .181 |  |  |  |  |
|  | **Education** | **.149** | **.061** | **.456** | **2.436** | **.021** |  |  |  |  |
|  | Sex* | .033 | .233 | .025 | .143 | .887 |  |  |  |  |
|  | FSRP | .020 | .020 | .204 | .983 | .334 |  |  |  |  |
| **Block 2** |  |  |  |  |  |  | **3.221** | **.365** | **.125** | **.020** |
|  | Age | .020 | .020 | .197 | .990 | .331 |  |  |  |  |
|  | **Education** | **.142** | **.057** | **.437** | **2.505** | **.018** |  |  |  |  |
|  | Sex* | .175 | .226 | .130 | .777 | .444 |  |  |  |  |
|  | FSRP | .027 | .019 | .271 | 1.392 | .175 |  |  |  |  |
|  | **Fornix MWF** | **14.858** | **6.341** | **.379** | **2.343** | **.026** |  |  |  |  |

Abbreviations: FSRP = updated Framingham Stroke Risk Profile (Dufouil et al., 2017); MWF = myelin water fraction

* Note males served as the reference group

Statistically significant (p < 0.05) results appear in bold font.

Table S2. Multiple Hierarchical Linear Regression Models for Association of Fornix MWF and Memory Functioning Adjusting for Demographics, Vascular Risk Burden, Hippocampal Volume, and Fornix DTI FA Among Cognitively Normal Participants (n = 34)

|  | Variable | B | *SE* | β | *t* | *p* | F | R^2^ | Δ R^2^ | Block p |
| --- | --- | --- | --- | --- | --- | --- | --- | --- | --- | --- |
| **Block 1** |  |  |  |  |  |  | 1.493 | .256 |  | .220 |
|  | Age | .037 | .026 | .362 | 1.419 | .168 |  |  |  |  |
|  | **Education** | **.143** | **.064** | **.439** | **2.234** | **.034** |  |  |  |  |
|  | Sex* | -.003 | .251 | -.003 | -.014 | .989 |  |  |  |  |
|  | FSRP | .019 | .023 | .189 | .816 | .422 |  |  |  |  |
|  | Hippocampal  Volume | .045 | .338 | .036 | .132 | .896 |  |  |  |  |
|  | Fornix DTI FA | 1.277 | 3.168 | .118 | .403 | .690 |  |  |  |  |
| **Block 2** |  |  |  |  |  |  | 2.105 | .371 | .115 | .081 |
|  | Age | .025 | .025 | .250 | 1.022 | .317 |  |  |  |  |
|  | **Education** | **.138** | **.060** | **.422** | **2.288** | **.031** |  |  |  |  |
|  | Sex* | .149 | .246 | .111 | .607 | .550 |  |  |  |  |
|  | FSRP | .025 | .022 | .253 | 1.155 | .259 |  |  |  |  |
|  | Hippocampal  Volume | -.019 | .319 | -.015 | -.059 | .954 |  |  |  |  |
|  | Fornix DTI FA | 1.115 | 2.972 | .103 | .375 | .711 |  |  |  |  |
|  | **Fornix MWF** | **14.398** | **6.746** | **.369** | **2.134** | **.043** |  |  |  |  |

Abbreviations: MWF = myelin water fraction; DTI = diffusion tensor imaging; FA = fractional anisotropy; FSRP = updated Framingham Stroke Risk Profile (Dufouil et al., 2017)

* Note males served as the reference group

Statistically significant (p < 0.05) results appear in bold font.

REFERENCES

Bondi, M. W., Edmonds, E. C., Jak, A. J., Clark, L. R., Delano-Wood, L., McDonald, C. R., . . . Salmon, D. P. (2014). Neuropsychological criteria for mild cognitive impairment improves diagnostic precision, biomarker associations, and progression rates. *J Alzheimers Dis, 42*(1), 275-289. doi:10.3233/jad-140276

Dale, A. M., Fischl, B., & Sereno, M. I. (1999). Cortical surface-based analysis. I. Segmentation and surface reconstruction. *Neuroimage, 9*(2), 179-194. doi:10.1006/nimg.1998.0395

Delis, D. C., Kramer, J., Kaplan, E., & Ober, B. A. (2000). *The California Verbal Learning Test-Second Edition*. New York: Psychological Corporation.

Deoni, S. C., Dean, D. C., O'Muircheartaigh, J., Dirks, H., & Jerskey, B. A. (2012). Investigating white matter development in infancy and early childhood using myelin water faction and relaxation time mapping. *Neuroimage*. doi:10.1016/j.neuroimage.2012.07.037

Dufouil, C., Beiser, A., McLure, L. A., Wolf, P. A., Tzourio, C., Howard, V. J., . . . Seshadri, S. (2017). Revised Framingham Stroke Risk Profile to Reflect Temporal Trends. *Circulation, 135*(12), 1145-1159. doi:10.1161/circulationaha.115.021275

Fischl, B., Salat, D. H., Busa, E., Albert, M., Dieterich, M., Haselgrove, C., . . . Dale, A. M. (2002). Whole brain segmentation: automated labeling of neuroanatomical structures in the human brain. *Neuron, 33*(3), 341-355.

Heaton, R. K., Miller, S. W., Taylor, M. J., & Grant, I. (2004). *Revised comprehensive norms for an expanded Halstead-Retain Battery: Demographically adjusted neuropsychological norms for African American and Caucasian adults scoring program*. Odessa, Florida: Psychological Assessment Resources.

Jak, A. J., Bondi, M. W., Delano-Wood, L., Wierenga, C., Corey-Bloom, J., Salmon, D. P., & Delis, D. C. (2009). Quantification of five neuropsychological approaches to defining mild cognitive impairment. *American Journal of Geriatric Psychiatry, 17*(5), 368-375. doi:10.1097/JGP.0b013e31819431d5

Jenkinson, M., Bannister, P., Brady, M., & Smith, S. (2002). Improved optimization for the robust and accurate linear registration and motion correction of brain images. *Neuroimage, 17*, 825-841.

Kongs, K. S., Thompson, L. L., Iverson, G. L., & Heaton, R. K. (2000). *Wisconsin card sorting test-64 card version (WCST-64)*. Odessa, FL: Psychological Assessment Resources.

Mori, S., Oishi, K., Jiang, H., Jiang, L., Li, X., Akhter, K., . . . Woods, R. (2008). Stereotaxic white matter atlas based on diffusion tensor imaging in an ICBM template. *Neuroimage, 40*(2), 570-582.

Smith, S. M., Jenkinson, M., Woolrich, M. W., Beckmann, C. F., Behrens, T. E., Johansen-Berg, H., . . . Matthews, P. M. (2004). Advances in functional and structural MR image analysis and implementation as FSL. *Neuroimage, 23 Suppl 1*, S208-219. doi:10.1016/j.neuroimage.2004.07.051

Stricker, N. H., Schweinsburg, B. C., Delano-Wood, L., Wierenga, C. E., Bangen, K. J., Haaland, K. Y., . . . Bondi, M. W. (2009). Decreased white matter integrity in late-myelinating fiber pathways in Alzheimer's disease supports retrogenesis. *Neuroimage, 45*(1), 10-16. doi:10.1016/j.neuroimage.2008.11.027
